# In Vivo Characterization of ONL1204, a Small Peptide Inhibitor of the Fas Receptor, as a Potential Neuroprotective Therapy for Geographic Atrophy and Dry Age-Related Macular Degeneration

**DOI:** 10.3390/biomedicines13092052

**Published:** 2025-08-22

**Authors:** Andrew J. Kocab, Marisol Cano, Marianna Bacellar-Galdino, Jeffrey A. Jamison, William J. Brock, David N. Zacks, James T. Handa

**Affiliations:** 1 ONL Therapeutics, Inc., Ann Arbor, MI 48104, USA; davzacks@umich.edu; 2 Wilmer Eye Institute, Johns Hopkins University, Baltimore, MD 21287, USA; mcano1@jhmi.edu; 3 Altasciences, Scott Township, PA 18411, USA; mbacellar-galdino@altasciences.com; 4 Ocular Consultants, Inc., Livonia, MI 48154, USA; jeff.jamison@ocular-consultants.com; 5 Brock Scientific Consulting, LLC, Hilton Head, SC 29926, USA; billbrock@comcast.net; 6 Kellogg Eye Center, Department of Ophthalmology and Visual Sciences, University of Michigan, Ann Arbor, MI 48105, USA

**Keywords:** age-related macular degeneration, geographic atrophy, neuroprotection, Fas, CD95, animal models

## Abstract

**Background**: Age-related macular degeneration (AMD) is a major cause of irreversible vision loss in the developed world, and the approved products for geographic atrophy (GA), a late-stage form of dry AMD, have shown limited efficacy and require frequent administration. Therefore, longer-lasting therapies with improved efficacy would be a welcome addition to AMD treatment. One potential therapeutic is ONL1204, a small peptide inhibitor of the Fas receptor that has prevented cell death and inflammation in retinal disease models. This study characterizes the pharmacokinetics (PK) and durability of protection conferred by ONL1204. **Methods**: Ocular pharmacokinetic profiles were generated over 3 months in rabbit and minipig following a single intravitreal (IVT) injection of ONL1204 at multiple doses. Ocular pharmacodynamics were evaluated in two models: a rabbit model using a single IVT injection of ONL1204 with a delayed sodium iodate challenge coupled with fluorescein angiography to quantify RPE loss, and a chronic mouse model that reflects key features of dry AMD disease pathology to assess the efficacy of repeat IVT administrations of ONL1204. **Results**: ONL1204 had prolonged residence in the ocular tissues of rabbit and minipig, with a vitreous humor half-life of over 100 days. ONL1204 demonstrated significant protection of the retinal pigment epithelium (RPE) in the rabbit sodium iodate model. In the chronic mouse model, two administrations of ONL1204 preserved RPE morphology, reduced caspase-8 activity, and decreased inflammation. **Conclusions**: These data represent key characteristics of ONL1204, highlighting its clinical potential as a therapeutic for chronic retinal diseases, including GA.

## 1. Introduction

Age-related macular degeneration (AMD) is a leading cause of irreversible vision loss among the elderly in the developed world [1,2]. Treatment with anti-vascular endothelial growth factor (VEGF) agents has provided impressive benefit to patients with neovascular or “wet” AMD. Conversely, for non-neovascular or “dry” AMD, the therapeutic options are limited. The C3 complement inhibitor pegcetacoplan and the C5 complement inhibitor avacincaptad pegol were approved by the United States Food and Drug Administration (FDA) in 2023 for the treatment of geographic atrophy (GA), a late-stage form of dry AMD. However, complement inhibition has so far demonstrated limited efficacy. Specifically, treatment with either agent reduced the rate of lesion growth by a modest 14–26% after one year of treatment [3,4]. Furthermore, these therapies require monthly or bi-monthly injections that result in an immense treatment burden for patients. Due to these limitations, efficacious and long-lasting therapies for this devastating and blinding disease remain a significant unmet medical need.

One potential therapeutic focus is the retinal pigment epithelium (RPE), as the dysfunction is a core driver of AMD pathobiology [5]. In AMD, the RPE degenerates, undergoing a morphological transformation that is associated with degradation of specialized functions that are essential for maintaining photoreceptor health and good vision [6,7,8]. Multiple signaling pathways contribute to this degeneration, ultimately leading to RPE cell death [9]. Among the cell death pathways, apoptosis becomes increasingly prominent with age, particularly in the macula [10]. Previous work has demonstrated that RPE cell death is exaggerated in AMD [11] and is most frequently seen at the edge of the atrophic lesion in patients with GA.

Apoptosis observed in the RPE is driven in part by the Fas receptor (CD95), a member of the tumor necrosis factor (TNF) superfamily of transmembrane receptors, that is at increased levels in donor eyes with AMD compared to controls [11]. Fas expression is also elevated in RPE cells in vitro and in vivo under oxidative stress, an AMD pathogenic factor [12,13,14]. Upon binding of Fas ligand (FasL, CD95L) to the receptor, Fas oligomerizes to recruit cytoplasmic factors that form the death inducing signaling complex (DISC), initiating the cascade of caspase activity that results in cell death [15,16,17]. The Fas activation can also activate the necroptotic pathway, another reported mechanism of RPE cell death in AMD, which is independent of caspase activity and is mediated through receptor interacting protein kinases (RIPKs) that activate membrane-damaging proteins and induce lytic cell death [5,18]. In addition to triggering cell death cascades, the pathologic activation of Fas in retinal disease promotes an inflammatory microenvironment through the induction and secretion of proinflammatory cytokines and chemokines that recruit inflammatory cells [19]. The Fas receptor, therefore, regulates important pathways that play well established and critical roles in AMD pathobiology.

Thus, the Fas receptor is a viable therapeutic modality that could reduce retinal and RPE cell inflammation and death during AMD. To this end, ONL Therapeutics, Inc. has developed a family of small peptides that inhibit Fas receptor activation. These peptides are based on a core 12-amino acid fragment of the Met protein, which has intrinsic Fas receptor blocking characteristics [20]. The lead compound, ONL1204, and a precursor molecule Met12 have been used successfully to reduce retinal cell death and inflammation in several preclinical models of retinal detachment, inherited retinal dystrophy, and glaucoma [18,21,22,23]. Furthermore, Met12 and ONL1204 treatment conferred protection in acute models of RPE toxicity induced by oxidative stress using either sodium iodate or cigarette smoke extract (CSE) [5,18]. Intravitreal administration of either of these peptides significantly reduces the downstream activation of both apoptotic and necroptosis pathways as well as reducing cytokine and chemokine production and the associated innate immune activation [18].

While the therapeutic benefit of targeting Fas has been shown previously, key questions remained around the utility of ONL1204 for use in chronic indications such as dry AMD and GA. In this study, we aimed to address these questions by characterizing the pharmacokinetics and durability of protection for ONL1204 in nonclinical in vivo models. The pharmacokinetics of ONL1204 was assessed in two animal species, rabbit and minipig, confirming an intravitreal half-life of approximately 100 days, which is notably long for a small peptide therapeutic. In addition, to determine whether the drug remained biologically active over time, we conducted studies to evaluate the durability of the protection conferred by ONL1204 in two models of RPE toxicity: a rabbit model using a single IVT injection of ONL1204 with a delayed sodium iodate challenge followed by assessment with fluorescein angiography to quantify RPE loss, and a chronic mouse model that reflects key features of dry AMD disease pathology with exposure to cigarette smoke and high-fat diet to characterize the efficacy of repeat IVT administrations of ONL1204. Rabbits were treated with ONL1204, followed by administration of sodium iodate at various time points after dosing. This design allowed us to assess whether ONL1204 retained sufficient activity to provide protection when the RPE were challenged up to two months after administration of the Fas inhibitor. We further tested the durability of protection conferred by ONL1204 in a chronic mouse model of RPE atrophy that uses exposure to cigarette smoke and high-fat diets to replicate aspects of the dry AMD phenotype. The results from these experiments support the conclusion that ONL1204 not only achieves sustained ocular exposure but also maintains protective efficacy over an extended period. Collectively, these data support the utility of ONL1204 as a therapeutic candidate for treating chronic retinal indications, including dry AMD and GA.

## 2. Materials and Methods

### 2.1. Animals and Care

All animal housing and care were performed in compliance with the United States Department of Agriculture (USDA) Guidelines with no other species kept in the same room.

Minipig studies were performed at Altasciences Preclinical Scranton, Inc. (Scott Township, PA, USA). Male Göttingen minipigs were purchased from Marshall BioResources (North Rose, NY, USA) and were housed individually. The study was approved by the Altasciences Institutional Animal Care and Use Committee (IACUC).

The rabbit pharmacokinetic study was performed at PharmOptima (Portage, MI, USA). Male Dutch-belted rabbits were purchased from Envigo Global Services, Inc. (Denver, PA, USA), and were individually housed. The study was approved by the IACUC at PharmOptima.

The rabbit sodium iodates (NaIO_3_) pharmacodynamic study was performed by Ocular Consultants (formerly Ophthy-DS, Portage, MI, USA). In the study, male Dutch-belted rabbits were purchased from Envigo Global Services, Inc. (Denver, PA, USA). Rabbits were individually housed in a social setting with enrichment. The study was approved by the IACUC at Western Michigan University.

For the mouse study, an equal number of male and female apolipoproteinB100 (apoB100) mice on a *C57/S129* background were used [24]. These animals carry a mutation in the editing gene *apobec1*, resulting in the predominant production of apoB100 in these mice, contrasting to the majority of mice that predominantly produce apoB48 [24]. For all mice strains, the *rd8* mutation was bred out. Mice were fed a standard diet until they were put in the smoking chamber and then were fed a high-fat diet (HFD) consisting of 60% fat, 20% protein, and 20% carbohydrate (Research Diets, Inc., New Brunswick, NJ, USA). The study was approved by the Johns Hopkins IACUC.

At the designated time points, rabbits and minipigs were euthanized by intravenous sodium pentobarbital (Akorn, Lake Forest, IL, USA, #59399-185-90), and mice were euthanized by cervical dislocation after isoflurane anesthesia. All studies were conducted according to the ARVO Statement for the Use of Animals in Ophthalmic and Vision Research.

### 2.2. ONL1204

ONL1204 is a 12-amino acid synthetic peptide Fas inhibitor. The peptide was provided in a proprietary vehicle at concentrations described below. The formulated peptide and the corresponding vehicle control were provided by ONL Therapeutics, Inc. (Ann Arbor, MI, USA).

### 2.3. Pharmacokinetic Study

In the pharmacokinetics rabbit study, animals received a single bilateral dose of ONL1204 at 2 mg/mL (100 µg/eye), 1 mg/mL (50 µg/eye), or 0.2 mg/L (10 µg/eye) in vehicle on Day 0 via a 50 µL IVT injection, with three animals per treatment group. Prior to injection, each eye was moistened with an ophthalmic Betadine solution, which was then washed out of the eyes with sterile saline. Then, 0.5% proparacaine was applied to the ocular surface prior to the injection.

For the minipig study, animals received unilateral IVT injections of 100 µL ONL1204 at 2 mg/mL (200 µg/eye) or 3 mg/mL (300 µg/eye), with 3 animals per treatment group. Prior to the IVT injection, a combination of 100 mg Telazol, 50 mg ketamine, and 50 mg xylazine per mL of solution was administered via intramuscular injection at 0.03–0.06 mL/kg. For two animals, dexmedetomidine (0.02 or 0.03 mg/kg) was administered as a supplementary anesthetic via intramuscular injection. Proparacaine was then administered to eye, followed by tropicamide. The area surrounding the eye was swabbed with an ophthalmic Betadine solution.

For both studies, animals were euthanized at the indicated time points. Eyes were enucleated, snap frozen in liquid nitrogen, and stored frozen until dissection for collection of vitreous, retina, and RPE/choroid for bioanalysis. Bioanalysis of ONL1204 concentration in the tissues was assessed by LC-MS/MS. For statistical determination, all values that were below the lower limit of quantification (LLOQ) were considered zero. Pharmacokinetic analysis was performed from the composite concentration versus time data generated for ocular tissue samples through Day 84 using Phoenix WinNonlin (version 8.5.2.4) software.

### 2.4. Sodium Iodate Challenge

A 20 mg/mL stock solution of sodium iodate (NaIO_3_, Sigma Aldrich, St. Louis, MO, USA, Cat#54007) was prepared in sterile 0.9% injectable saline (Cytiva, Marlborough, MA, USA, Z1376), aliquoted into single-use vials, and stored at −20 °C until use. On the day of dosing, aliquots were thawed at room temperature and used immediately. For NaIO_3_ administration, rabbits were weighed and anesthetized with 2–5% isoflurane (Dechra, Norwich, United Kingdom, 17033-094-25) delivered in medical-grade oxygen at a flow rate of 1.0 to 1.5 L/min via chamber until sedated, then moved to facemask for the injection procedure. The left ear was shaved and disinfected with 10% alcohol prior to intravenous injection. An indwelling catheter was inserted into the marginal ear vein and flushed with sterile 0.9% injectable saline to ensure patency. Sodium iodate was administered intravenously at a dose of 18 mg/kg body weight, followed by a 0.3 mL saline flush. This dosing paradigm, delivered via the marginal ear vein, reliably induces bilateral retinal lesions of comparable size in both eyes of male Dutch-belted rabbits allowing each animal to serve as its own control. Ten animals were used in each treatment group and 6 animals served as the naïve control group.

### 2.5. Fluorescein Angiography

Fluorescein angiography was performed following topical anesthesia of the rabbit eyes with proparacaine hydrochloride 0.5% (Akorn, 24208-730-06) and pharmacologic dilation with tropicamide (Akorn, 17478-102-12) and phenylephrine (Akorn, 17478-206-12). The rabbits were anesthetized with isoflurane and the ear was prepared as stated above. A 50 µL bolus of 10% sodium fluorescein (Akorn, 59399-001-04) was injected into the marginal ear vein and allowed to circulate for at least 2 min prior to imaging. Fundus images were captured (Spectralis, Heidelberg Engineering) to document and quantify the extent of visible choroidal and RPE degeneration in both eyes. The area of RPE depigmentation was quantified using ImageJ software (version 1.51j8) [25]. In certain cases where the damage extended well into the periphery it was not possible to image the entire edge of the affected area, and these cases were labeled “too large to grade” and were excluded from the final analysis.

### 2.6. Smoke Exposure

At 8 weeks of age, male and female mice were placed in a smoking chamber for 5 h/day, 5 days/week for 6 months following our published protocol [26]. Briefly, smoke is delivered to the chamber by a machine (Model TE-10, Teague Enterprises, Davis, CA, USA) burning 8 cigarettes (2R4F reference cigarettes (2.45 mg nicotine/cigarette; Tobacco Research Institute, University of Kentucky) at a time [26]. Smoke puffs are of 2 s in duration, flowing at a rate of 1.05 L/min. This provides a standard puff of 35 cm^3^ and a total of 8 puffs per minute. The machine produces side stream (89%) and mainstream smoke (11%), and the chamber atmosphere is monitored to maintain total suspended particulate at 90 mg/m^3^, and carbon monoxide at 350 ppm [26]. An intravitreal injection of ONL1204 (ONL Therapeutics, Ann Arbor, MI) or vehicle was given 3.5 and 5 months after initiation of cigarette smoke.

### 2.7. Immunohistochemistry and Microscopy

For retinal and RPE flatmount immunofluorescence confocal microscopy, mice were euthanized and eyes enucleated (7 eyes/treatment group) to be processed for staining for IBA1 (microglia/monocyte marker) and ZO-1 (RPE morphology). The anterior segments were removed, and the retina was carefully separated from the RPE/choroid complex. Tissues were fixed in 2% paraformaldehyde in Tris-buffered saline (TBS) at 4 °C overnight. Retina flatmounts were processed with TBS/1% bovine serum albumin (BSA) and 5% donkey serum overnight at 4 °C. The flatmounts were then incubated with goat polyclonal anti-AIF-1/IBA1 antibody (1:50; Novus biologicals, Centennial, CO, USA) for 48 h at 4 °C, washed, and incubated with donkey anti-goat Cy3 secondary antibody (Abcam, Cambridge, MA, USA) overnight at 4 °C [27]. To process the RPE/choroid flatmounts, samples were covered with 5% normal donkey serum (Jackson ImmunoResearch Inc., West Grove, PA, USA) at 4 °C overnight, washed in 0.1% Triton X-100 in TBS, and then incubated at 4 °C overnight with a primary antibody mouse monoclonal anti-ZO-1 (1:100, cat. #339194; Thermo Fisher Scientific, Waltham, MA, USA) [27]. After washing, four pie cuts were made in the retina and RPE/choroid to flatten the samples [27]. Flatmounts were imaged with a confocal microscope (ZEN LSM 710, Carl Zeiss microscopy, Thornwood, NY, USA) collecting Z-stack tiles of the entire sample. For image processing, the images were divided into 4 quadrants. For retinal quadrants, microglia/monocytes/macrophages, defined by IBA1-immunolabeled cell bodies, were counted in five 20 × power fields per flatmount. For RPE/choroid quadrants, each image was used to measure the RPE cell aspect ratio and the average size per cell using ImageJ after setting a threshold as indicated by the software At least 15 cells were measured/experimental group, with n = 5 animals/group, using our previously published protocol [27].

### 2.8. Caspase-8 Activity Assay

After the retina and RPE/choroid were harvested, protein was extracted using RIPA Buffer (Sigma-Aldrich, St Louis, MO, USA) with Proteinase inhibitor and phosphatase inhibitor. Nine eyes per treatment group were used in this assay, and each sample included 75 μg of protein to standardize the amount of tissue with the Luminescent Caspase-Glow 8 assay kit (Promega, Madison, WI, USA) used according to manufacturer’s protocol. RPE cell death was determined by normalizing the measured luminescence by the total RPE cell count.

## 3. Results

### 3.1. ONL1204 Pharmacokinetics

**Figure 1:**
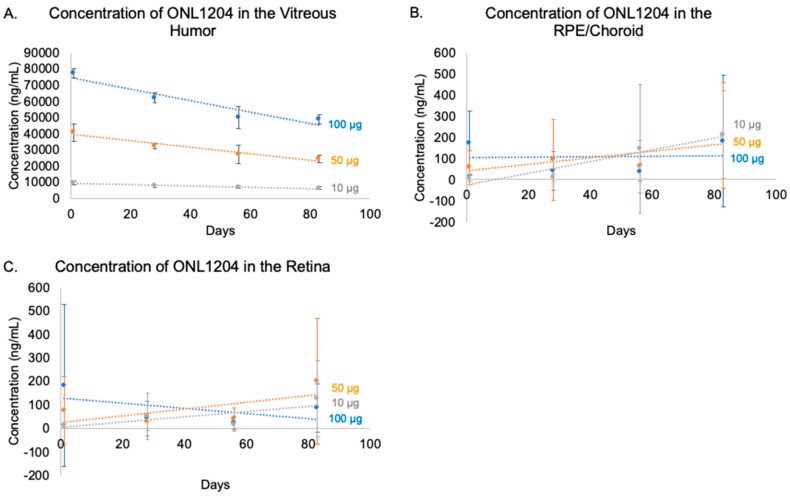
Concentrations of ONL1204 in rabbit ocular tissues over three months. ONL1204 was administered by IVT injection to Dutch-belted rabbits at 10, 50, or 100 µg/eye. Ocular tissues were collected on Days 1, 28, 56, and 83. (**A**) ONL1204 is slowly released from the vitreous humor. ONL1204 reaches the target tissue and is detectable in the retina (**B**) and RPE/choroid (**C**). Error bars represent standard deviation; n = 6 eyes per group per time point.

A suitable pharmacokinetic profile is a major requirement for therapy intended for a chronic disease. The concentration of ONL1204 was measured in the Dutch-belted rabbit over three months to generate a pharmacokinetic profile. Following the administration of a single IVT injection of 10, 50, or 100 µg/eye of ONL1204, the Fas inhibitor was detected in the vitreous humor, RPE/choroid, and retina (Table 1). Consistent with a profile supporting chronic intervention, ONL1204 was present in the vitreous humor throughout the entire 83-day study, exhibiting a clear dose-dependent initial concentration and a consistent, slow time-dependent clearance across all doses, reflected in calculated vitreous half-lives exceeding 100 days (147, 116, and 124 days for 10, 50, and 100 µg doses, respectively). This gradual vitreal elimination is consistent with the observed prolonged detection in posterior tissues. However, while ONL1204 was also detectable in the retina and RPE/choroid, concentrations in these tissues were substantially lower and exhibited higher levels of inter-animal variability compared to the vitreous (Figure 1, Table 1), with standard deviations frequently exceeding mean values. This pronounced variability complicates the interpretation of dose–response relationships and kinetic patterns in these tissues. Although the data suggest a potential trend for gradual transfer or accumulation over time in the RPE/choroid (particularly at the 10 µg dose) and a late increase in retinal concentration (10 µg and 50 µg doses by Day 83), supporting the concept of prolonged release from the vitreous reservoir, the high variability and missing standard deviations necessitate caution in definitively characterizing tissue-specific pharmacokinetics.

**Figure 2:**
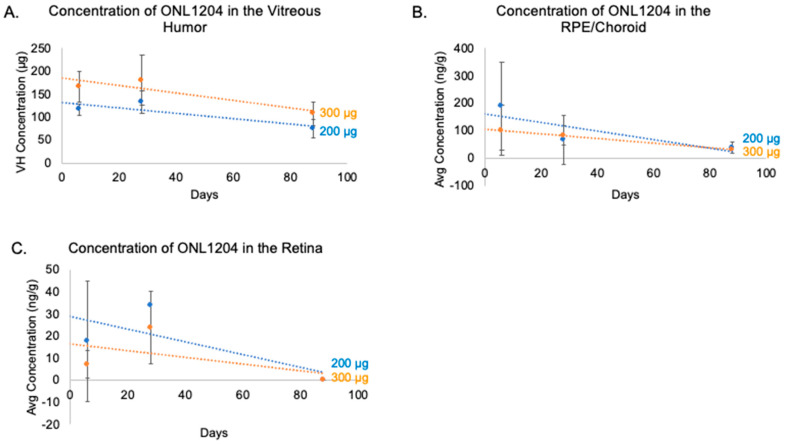
Concentrations of ONL1204 in minipig ocular tissues over three months. ONL1204 was administered by IVT injection to Göttingen minipigs at 200 or 300 µg/eye. Ocular tissues were collected at Days 6, 28, and 88. (**A**) ONL1204 exhibits a slow release from the vitreous humor. (**B**) ONL1204 concentrations in the retina. (**C**) ONL1204 is detectable in the RPE/choroid. Error bars represent standard deviation; n = 3 eyes per group per time point.

To further explore the ocular pharmacokinetics of ONL1204, a 3-month pharmacokinetic profile for ONL1204 was conducted in Göttingen minipigs. The minipig has become a more commonly used species in ocular studies, complementing the use of rabbit due to a more similar anatomy with the human eye, including size and vitreous volume [28]. ONL1204 was administered by IVT injection on Day 0 at a dose of either 200 or 300 µg/eye. ONL1204 was detected in the RPE/choroid, retina, and vitreous humor (Table 2). The average ONL1204 levels in the vitreous humor ranged from 75 to 180 µg and were directly proportional to the dose injected. Interestingly, the amounts detected in the vitreous at Day 6, the earliest time point tested, were substantially less than the administered dose (Figure 2A). This drop may be attributable to the sample preparation and extraction method used during the bioanalysis. Alternatively, this result may suggest a two-phase distribution pattern, where a bolus of the drug migrates quickly to the retina following administration while the remaining the drug forms a depot and distributes gradually. ONL1204 was detected in the vitreous at all time points. ONL1204 was also detected at all time points in the RPE/choroid, with an average concentration that ranged from 30 to 189 ng/g. (Figure 2B). For the retina, the average ONL1204 concentrations ranged from 2 to 24 ng/g (Figure 2C). ONL1204 was detected at all time points except at day 88, where the retinal samples were below the lower limit of quantification (LLOQ = < 1 ng/mL). The half-life values for each dose level of ONL1204 were calculated from the vitreous humor data. The 200 µg/eye dose had a half-life of 112.17 days, while the 300 µg/eye dose had a half-life of 117.18 days. Collectively, these data indicate that the pharmacokinetic profile of ONL1204 is suitable for use in a chronic retinal disease such as AMD with extended intervals between injections.

### 3.2. ONL1204 Pharmacodynamics

**Figure 3:**
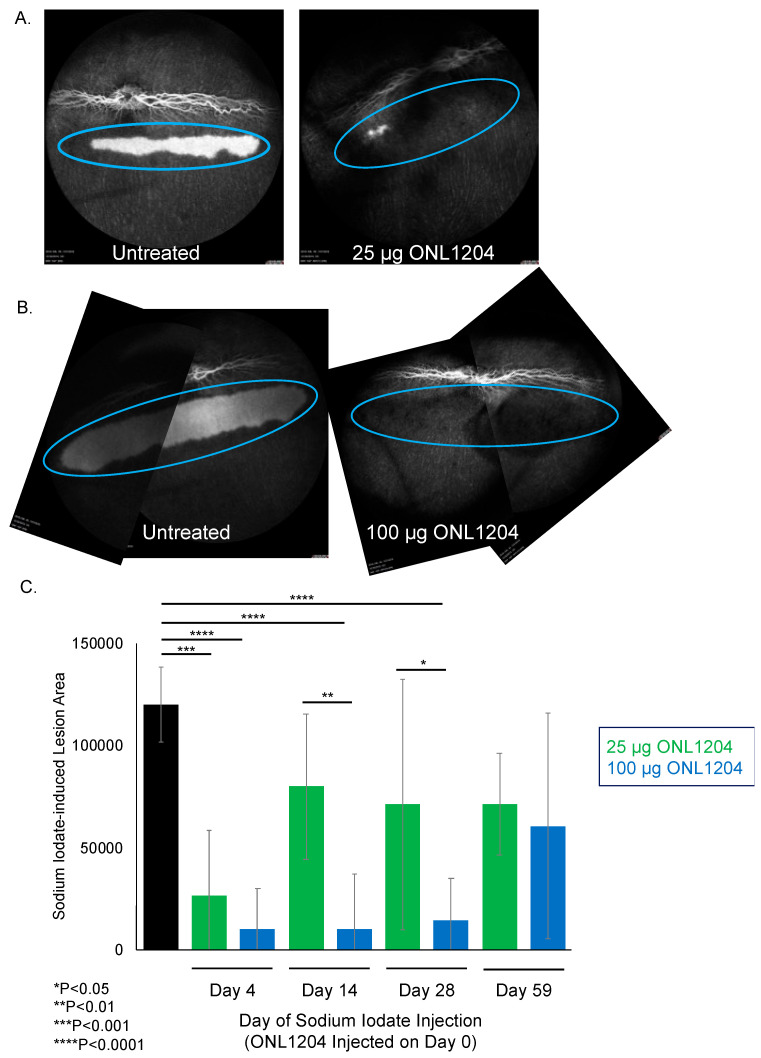
ONL1204 confers sustained protection of RPE in a delayed sodium iodate insult rabbit model. ONL1204 was given by IVT on Day 0 to one eye of each animal and the opposite eye was left untreated. Sodium iodate was administered on the indicated days. (**A**,**B**) show representative fluorescein angiography images of untreated and 25 or 100 µg ONL1204-treated eyes following sodium iodate challenge on Day 28. The blue ovals highlight the area of RPE loss and the “windowing” effect observed during the fluorescein angiography. (**C**) Quantitative assessment of sodium iodate-induced lesion size. A 1-way ANOVA with Dunnett’s post hoc multiple comparison of treated eyes to naïve control was used as the statistical method with a 0.05 *p*-value cutoff. * *p* < 0.05, ** *p* < 0.01, *** *p* < 0.001, **** *p* < 0.0001.

With a favorable pharmacokinetic profile, we next evaluated the durability of ONL1204 protection. We previously demonstrated that Fas inhibition can protect against acute oxidative stress [5,18]. To assess the durability of the ONL1204 protective effect, we employed the sodium iodate model using a delayed challenge paradigm. In these experiments, a single intravitreal (IVT) injection of either 25 µg or 100 µg ONL1204 was administered on Day 0, and sodium iodate was delivered systemically on Day 4, 14, 28, or 59. The dose of the systemic sodium iodate had been selected based on a titration that identified a level of systemic sodium iodate that led to localized and quantifiable damage to the rabbit RPE. This damage was assessed one day after sodium iodate administration by quantifying the depigmented lesion area, defined as regions of visible choroid (“window defect”) on fluorescein angiography (Figure 3A,B). The protective effect of ONL1204 in the treated eye was compared to the contralateral eye, which received vehicle alone (0 µg ONL1204).

When sodium iodate was administered four days after ONL1204 injection, both the 25 µg and 100 µg treatment groups exhibited comparable levels of protection, with no statistically significant difference in lesion size between the two doses (Figure 3C), which suggests that these doses are similarly effective when the oxidative insult occurs shortly after treatment. In contrast, when sodium iodate was administered 14 or 28 days post-injection of ONL1204, animals receiving 100 µg ONL1204 exhibited significantly smaller lesion areas compared to both the vehicle-treated controls and the 25 µg group, indicating the larger dose provides protection for a longer time. When the sodium iodate challenge was delayed to 59 days post-treatment, no differences were observed between ONL1204-treated and control eyes despite a 40% and 50% reduction in mean lesion areas in the 25 µg and 100 µg groups, respectively, relative to vehicle controls (*p* = 0.1106; Figure 3C).

**Figure 4:**
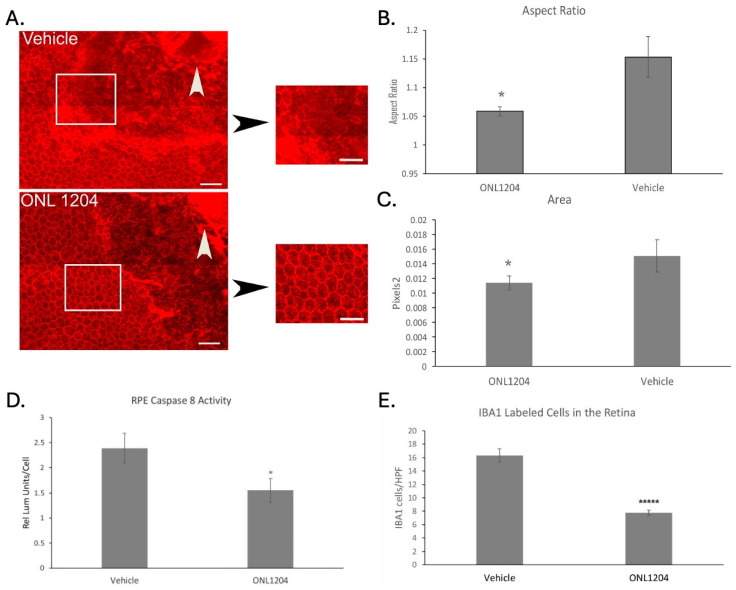
ONL1204 mitigates RPE degeneration in a chronic mouse model of dry AMD. RPE flatmounts were collected after apoB100 mice were exposed to 6 months of CS/HFD and given two IVT injections of ONL1204 6 weeks apart. (**A**) These flatmounts were immunolabeled with ZO1 antibody to visualize the RPE cell margin using confocal microscopy. The dysmorphic RPE with a variety of cell shapes ranging from hexagonal to elongated in the central fundus. Note intracytoplasmic staining for ZO1 in dysmorphic cells. Bar = 25 μm; n = 7 eyes per group. The cobblestone morphology is preserved in ONL1204-treated mice relative to vehicle-treated mice in the same central region. White box outlines the magnified images of the RPE. White arrowhead points to optic nerve. Bar = 25 μm. (**B**,**C**) The aspect ratio and cell area of total RPE are reduced by ONL1204 treatment. * *p* < 0.05. n = 7 eyes per group. (**D**) The RPE of apoB100 mice exposed to HFD/CS and given two intravitreal injections of ONL1204 had decreased caspase-8 activity compared to vehicle injected mice. * *p* < 0.05. n = 9 eyes per group. (**E**) Retinal flatmounts were immunolabeled with IBA1 antibody to visualize immune cells using confocal microscopy, showing the decrease in IBA1 labeled cells, as counted in five high-power fields. ***** *p* < 0.000001. n = 7 eyes per group. Statistical analysis was performed using a two-sample *t*-test assuming unequal variances.

These data indicate that ONL1204 can confer sustained protection to RPE against delayed induced acute oxidative toxicity. We next examined the effect of ONL1204 in a more pathophysiologically relevant model of chronic oxidative stress. The apoB100 mouse strain contains a mutation in the editing gene *apobec1*, so that these animals predominantly produce apoB100, the backbone of lipoproteins in humans, in contrast to most mice that predominantly produce apoB48, the backbone of chylomicrons [24]. Given that apoB100 lipoproteins accumulate in Bruch’s membrane in AMD, these mice are relevant for studying AMD pathobiology related to lipid metabolism. When fed a high-fat diet (HFD) and exposed to daily cigarette smoke (CS) for 6 months, these mice develop a dAMD-like phenotype [26]. Specifically, apoB100 mice develop patches of posterior RPE degeneration, which correspond with dysmorphic RPE and RPE loss observed on flatmounts immunolabeled with ZO1 (Figure 4A). ONL1204 treatment helped to protect the posterior RPE morphology, as shown in ZO1 immunolabeled RPE flatmounts (Figure 4A). The posterior RPE of ONL1204-treated mice had cell aspect ratio and cell area calculations that were comparable to posterior RPE from WT mice [27] and were significantly lower than the control, vehicle-treated RPE (Figure 4B,C). To confirm the Fas-inhibitory effect of ONL1204 in this model, we measured the activity of caspase-8, the first downstream target of the activated Fas receptor. Caspase-8 activity in whole RPE lysates of mice exposed to chronic CS/HFD and treated with ONL1204 was significantly reduced relative to eyes from CS/HFD exposed mice treated with vehicle alone (Figure 4D). Since Fas signaling also activates the retinal innate immune response that recruits inflammatory cells [19], the impact of ONL1204 treatment on innate immune cell activation in the retina was evaluated. Fas inhibition with ONL1204 treatment decreased IBA1+ inflammatory cell infiltration in the retina as compared to vehicle treated mice (Figure 4E). These data are consistent with Fas signaling inhibition by ONL1204 that both preserves RPE morphology and reduces the inflammatory microenvironment in this dry AMD model.

## 4. Discussion

In early 2023, the complement C3 inhibitor, pegcetacoplan (Syfovre, Apellis Pharmaceuticals, NDA 217171), was approved by the FDA for the treatment of the advanced stage of AMD, or GA, which was followed soon after by the approval of the complement C5 inhibitor avacincaptad pegol (Izervay, Astellas, NDA 217225). Both of these agents were shown to reduce the rate of GA lesion growth relative to sham treatment. However, the reduction in the rate of lesion growth by both agents is modest, from between 14 and 26% at one year of treatment, with better effect seen with monthly injection [3,4]. Notably, vision does not improve with either agent, despite slowing GA lesion growth. In addition to the significant treatment burden of monthly or every-other-month injection, the development of coincident neovascular AMD increases over time, with resultant worse vision, raising substantial safety concerns. These results suggest that effective treatment for patients with GA remains a large unmet need.

The efficacy of treatment is likely to be increased by mitigating more than one pathogenic pathway. One potential target for treating GA is the Fas receptor, a key mediator of cell death and inflammation, which are both prominent pathogenic factors in AMD. Fas-mediated cell death and inflammation are seen in other ocular diseases including retinal detachment (RD), inherited retinal degeneration (IRD), and glaucoma [21,23,29]. For AMD and GA, Fas expression was found to be elevated in the RPE of post-mortem AMD eyes [11]. In rabbits, Fas inhibition using Met12 preserved the structure and viability of the RPE following sodium iodate challenge [18]. The influence of Fas on the intrinsic pathway may be beneficial for treatment in AMD given that mitochondrial dysfunction, which initiates the intrinsic apoptotic pathway, is a well-established factor in AMD pathogenesis [30,31,32].

ONL1204, a 12-amino-acid peptide Fas inhibitor, is currently under development for the treatment of multiple retinal diseases and conditions. Treatment with ONL1204, or the analog Met12, has been shown to protect a range of retinal cells, including photoreceptors, RPE, and retinal ganglion cells (RGC), across a gamut of retinal disease models [5,18,21,22]. Furthermore, Fas inhibitor treatment also downregulates the inflammatory phenotype associated with disease progression, including reduced microglia/macrophage activation and recruitment and decreased inflammatory gene expression, including the complement genes [5,18,21,22] ONL1204 has been shown to be safe in a Phase 1 clinical trial in retinal detachment (NCT03780972) and recently completed a Phase 2 clinical trial (NCT05730218) in that indication. Additionally, ONL1204 has been tested in Phase 1b studies in patients with GA (NCT04744662) and open-angle glaucoma (NCT05160805). In all of these cases, ONL1204 has demonstrated its potential to preserve retinal cells and improve function. A Phase 2 study testing ONL1204 in patients with GA is currently being initiated (NCT06659445).

The results presented in the current study support the use of ONL1204 as a potential treatment in chronic retinal diseases and conditions, with the overall pharmacokinetics profile supporting the potential for every 3- to 6-month dosing. The bioanalysis of the ocular tissues showed that ONL1204 levels gradually decline over time. The detectable amounts of ONL1204 in the retina and RPE/choroid were variable, which may have been due, at least in part, to variable penetration of the drug through the inner limiting membrane (ILM), an observation often associated with gene therapies and hypothesized for other therapeutic modalities [33,34,35]. Alternatively, the RPE/choroid and retina may have features that sequester the drug from our current bioanalytical detection methods. The presence of ONL1204 in the choroid was, however, interpreted to indicate drug flux through the target tissues. The presence of ONL1204 in the RPE/choroid was not due to exposure through systemic circulation. The peptide is rapidly broken down by peptidases in the plasma, and no ONL1204 has been detected in the plasma following IVT administration. The variability in the retention time across the retinal tissues may be due to multiple causes. We have hypothesized that ONL1204 along with the excipients in the vehicle may be interacting with components in the vitreous humor, such as hyaluronic acid. This interaction may result in the slow and gradual release of ONL1204 from the vitreous. In contrast, ONL1204 may exhibit a different observable retention time the retina and RPE/choroid due to processing of the peptide by peptidases or cellular uptake and adhesion, and these factors may differ between rabbit and minipig.

The current study also illustrated the durable protection conferred by ONL1204 in two animal models of RPE atrophy. In the rabbit sodium iodate model, ONL1204 provided significant protection out to at least one month. At two months, protection of the RPE was observed, but did not reach statistical significance. This may be due to the aggressive oxidative toxicity of sodium iodate on RPE cells. It is likely that the oxidative stress level seen in clinic is substantially less aggressive than sodium iodate, potentially translating to a more prolonged protection following ONL1204 treatment. The large error bars seen in this study were likely due to variances in the induction of damage, as the sodium iodate model, as applied here, was variable in inducing consistently, measurable RPE cell death. In this model, both doses of ONL1204 appeared to provide similar levels of protection on Day 4, but greater differences in efficacy were seen between the doses at later time points. The underlying rationale for this observation is not clear. However, one hypothesis is that a bolus of drug quickly migrates to the back of the eye following administration, saturating the tissue with inhibitor, regardless of dose. The remaining drug forms a depot in the vitreous humor, which slowly migrates to the back of the eye. The lower dose may not be able to sufficiently supply the retina with ONL1204 to maintain the protective threshold, leading to the dose-dependent responses. Subsequent studies may continue to explore this observation and generate a better understanding of the pharmacodynamics of the Fas inhibitor.

For the chronic mouse model, ONL1204 was administered six weeks apart and helped to maintain a healthy appearance of the RPE, reduced caspase-8 activity, and decreased microglia activation. While a dosing frequency longer than six weeks may have been possible in this mouse model, this was not tested in the present study, as it was unclear how well the pharmacokinetics data from the larger eye species (rabbit and minipig) would translate to the mouse eye. Future work may expand the testing of ONL1204 in the chronic mouse model to better understand the pharmacokinetics and efficacy relationship across species. Furthermore, these studies would provide an opportunity to continue to explore the mechanistic implications of Fas inhibition in a chronic setting and continue to characterize the impact on the RPE, photoreceptors, and other cell types in the retina. The durability and pharmacokinetics data support the continued development of ONL1204 as a treatment for a chronic retinal disease such as dry AMD.

The RPE can die by mechanisms other than apoptosis, such as necroptosis [36,37]. Fas has been shown to mediate these other cell death mechanisms, including necroptosis. Importantly, we and others have previously demonstrated that blocking Fas can prevent intracellular shunting between these death mechanisms [18,38]. Additionally, Fas has been associated with autophagy [39]. While autophagy mediates proapoptotic signals and impaired autophagy is a known contributing factor in AMD [40], it is unclear whether impairing autophagy by Fas inhibition will reduce its therapeutic potential. While we did not evaluate autophagy in our studies, it would be a valuable future research direction. The RPE may undergo additional alterations during AMD progression, including epithelial–mesenchymal transition (EMT) and transdifferentiation [41,42,43,44,45,46]. While the results presented in the current study focus on assessing the PK and durability of ONL1204, future work could continue to explore the potential impact of Fas inhibition on these RPE changes. We also recognize the limitations of our experimental design. In the chronic mouse study, ONL1204 was not fully therapeutic since we first administered the Fas inhibitor when mice start to develop recognizable pathologic changes. Similarly, in the rabbit durability study, ONL1204 was administered prior to the sodium iodate challenge. Therefore, it is unknown how effective ONL1204 will be when administered with more advanced disease stages. However, the favorable pharmacokinetic profile of ONL1204 suggests that dosing intervals longer than the monthly and bimonthly injections of the current complement inhibitors may be therapeutic. If so, this could help reduce patient burden.

In summary, our data suggest that Fas receptor inhibition is a promising therapeutic option for reducing RPE cell death and retinal inflammation in the patients with dry AMD and support the continued development of ONL1204 for this indication.

## Figures and Tables

**Table 1:** Average concentration of ONL1204 (±standard deviation) in the ocular tissues of Dutch-belted rabbits following a single intravitreal injection on day 0.

Tissue	Day	10 µg/Eye ONL1204	50 µg/Eye ONL1204	100 µg/Eye ONL1204
Vitreous Humor (µg) ^1^	1	9.3 ± 1.2	40.6 ± 5.2	77.1 ± 2.7
	28	7.7 ± 1.0	32.5 ± 1.7	62.0 ± 3.2
	56	6.9 ± 0.8	27.1 ± 5.9	50.0 ± 6.7
	83	6.2 ± 0.8	24.5 ± 2.4	48.7 ± 3.0
Retina (ng/g) ^2^	1	11.2 ± 8.4	75.6 ± 144.0	183.0 ± 346.0
	28	51.0 ± 97.9	27.0 ± 36.6	42.3 ± 72.1
	56	14.3 ± 15.1	37.8 ± 48.4	22.7 ± 27.9
	83	125.0 ± 162.0	201.0 ± 268.0	86.1 ± 103.0
RPE/Choroid (ng/g) ^3^	1	5.45 ^4^	58.0 ± 79.8	174.0 ± 149
	28	10.5 ^4^	92.4 ^4^	40.6 ^4^
	56	144.0 ^4^	61.8 ^4^	36.2 ± 41.0
	83	213.0 ± 207.0	209.0 ± 251.0	182.0 ± 312.0

^1^ Vitreous humor LLOQ = 0.1 µg; ^2^ retina LLOQ = 6 ng/g; ^3^ RPE/choroid LLOQ = 15.0 ng/g; ^4^ insufficient data for determination of standard deviation.

**Table 2:** Average concentration of ONL1204 (±standard deviation) in the ocular tissues of Göttingen minipigs following a single intravitreal injection on day 0.

Tissue	Day	200 µg/Eye ONL1204	300 µg/Eye ONL1204
Vitreous Humor (µg) ^1^	6	116.0 ± 12.7	166.0 ± 34.0
	28	132.0 ± 24.5	180.0 ± 54.1
	88	74.9 ± 20.6	108.0 ± 25.5
Retina (ng/g) ^2^	6	17.8 ± 27.3	7.20 ± 6.37
	28	2.05 ^4^	23.8 ± 16.4
	88	<LLOQ	<LLOQ
RPE/Choroid (ng/g) ^3^	6	189.0 ± 160.0	101.0 ± 91.4
	28	66.5 ± 87.8	82.4 ± 34.5
	88	38.2 ± 21.7	29.8 ^4^

^1^ Vitreous humor LLOQ = 0.1 µg; ^2^ retina LLOQ = 2.5 ng/g; ^3^ RPE/choroid LLOQ = 20.0 ng/g; ^4^ insufficient data for determination of standard deviation.

## Data Availability

The data presented in this study are available on reasonable request from the corresponding author.

## Abbreviations

The following abbreviations are used in this manuscript:

AMD	Age-related macular degeneration
VEGF	Vascular endothelial growth factor
GA	Geographic atrophy
PK	Pharmacokinetics
IVT	Intravitreal
RPE	Retinal pigment epithelium
FDA	United States Food and Drug Administration
TNF	Tumor necrosis factor
DISC	Death-inducing signaling complex
NaIO_3_	Sodium Iodate
RIPK	Receptor-interacting protein kinase
CSE	Cigarette smoke extract
USDA	United States Department of Agriculture
IACUC	Institutional Animal Care and Use Committee
apoB100	apolipoproteinB100
HFD	High-fat diet
LC-MS/MS	Liquid chromatography-mass spectrometry/mass spectrometry
TBS	Tris-buffered saline
BSA	Bovine serum albumin
AIF-1/IBA1	Allograft inflammatory factor 1/ionized calcium-binding adapter molecule 1
ZO-1	Zonula occludens-1
RIPA	Radioimmunoprecipitation assay buffer
LLOQ	Lower limit of quantification
CS	Cigarette smoke
WT	Wild type
RD	Retinal detachment
IRD	Inherited retinal degeneration
RGC	Retinal ganglion cell
ILM	Inner limiting membrane
EMT	Epithelial–mesenchymal transition
